# Assessment of the effect of paclobutrazol treatment on the activity of genes related to flavonoid synthesis in *Brassica napus* under salt stress conditions

**DOI:** 10.1186/s12870-026-08736-y

**Published:** 2026-05-20

**Authors:** Alireza Mirzaei, Jamshid Fooladi, Bahman Fazeli-Nasab

**Affiliations:** 1 Department of Biotechnology, Faculty of Biological Sciences, Alzahra University, Tehran, Iran; 2 Department of Agronomy and Plant Breeding, Agriculture Institute, Research Institute of Zabol, Zabol, Iran

**Keywords:** Paclobutrazol, Salt stress, Hydropathicity, Flavonoids, Rapeseed

## Abstract

The samples were under the influence of the treatment with pyrrolizine.

Salinity adversely impacts oilseed production by leading to excessive salt accumulation and degrading soil structure. In rapeseed, salinity specifically hinders germination and the growth of aerial organs while also reducing the production of flavonoids and anthocyanins. Recent research has demonstrated that paclobutrazol treatment can enhance cellular activities under environmental stress. Accordingly, this study investigated the effects of paclobutrazol on the molecular mechanisms related to the synthesis of flavonoids, as one of the most important natural polyphenolic compounds. In this study, after applying salinity stress, the samples were under the influence of the treatment with paclobutrazol. The results of the expression evaluation of flavonoid synthesis-related genes production (MYB111 and MYB12) showed that paclobutrazol treatment had a significant effect on increasing the expression of these genes. The findings of this study also indicated that paclobutrazol treatment, in addition to reducing the negative effects of salinity, significantly increased the production of anthocyanins and the growth of plant aerial organs. MDA measures membrane damage from stress. SOD and POD are key antioxidant enzymes that protect plant cells from oxidative harm. Bioinformatics analyses of this study also showed that MYB111 and MYB12 genes have negative hydropathicity while interacting with a network of genes and transcription factors. These findings indicate that these genes, in addition to creating resistance to salinity stress, are also effective in reducing the effects of drought stress. This study's results indicate that applying paclobutrazol to oilseeds effectively prevents damage from salinity stress and reduces sodium ion accumulation in the plant structure.

## Introduction

Salinity stress poses a significant challenge to agricultural production globally, threatening food security. It not only damages the physiological and biochemical mechanisms within plants but also disrupts various processes essential for plant growth and development [1]. When the amount of solutes in the root activity area increases, water absorption is disturbed [2]. Electrical conductivity of 4 dS/m is the boundary of separating saline soils from non-saline soils [3]. Saline soils contain soluble salts, including chlorides, sulfates, sodium bicarbonates, magnesium, and calcium [4]. In saline soils, in addition to the increase in osmotic pressure caused by the accumulation of soluble salts, the toxicity of ions such as sodium, chlorine, and boron is also a concern [5]. The imbalance between evaporation, transpiration, and rainfall in these regions results in the accumulation of soluble salts in the soil. After periods of water stress, the concentration of these salts becomes a major limiting factor for plant growth. Key sources of salts in saline soils include precipitation, the decomposition of primary and secondary minerals, residual salts from ancient marine or lake sediments, fossil salts, rising groundwater tables, and improper fertilization. Salinity disrupts several processes: it hinders the transfer of nutrients from the soil to the plant, impedes the release of elements from the solid state into the soil solution, affects the movement of nutrients toward the roots, and interferes with the plant's ability to absorb and utilize these elements in various organs [6, 7]. Saline soils also require more energy from the plant to absorb water and regulate biochemical activities, which ultimately hurts plant growth. Today, emerging technologies such as genetic engineering, along with the use of markers and probes, have had a significant impact on molecular modification and dynamic tracking of plant growth under salt stress conditions. These developments represent a significant step in understanding the processes related to salinity stress and its control [8].

Paclobutrazol, as a plant growth regulator, plays an important role in plant reproductive growth. This substance limits the production of this hormone by inhibiting the biosynthesis pathway of gibberellic acid and is therefore known as a vegetative growth inhibitor [9–11]. In addition, paclobutrazol has a significant effect on reducing environmental stresses in plants, as research has shown that the resistance of plants such as peach, grape, pear, and other species to various environmental stresses increases with the use of this substance [12]. In 2025, Hajihoseinlou et al. reported that paclobutrazol enhances antioxidant activity, especially that of superoxide dismutase, in *Triticum aestivum*. Furthermore, paclobutrazol helps plants cope with salt stress by reducing the absorption and accumulation of chloride and sodium ions in their tissues. Overall, the application of paclobutrazol minimizes membrane damage, increases cellular water content, improves photosynthesis rates, and elevates the concentrations of essential pigments such as chlorophyll and carotenoids under salt stress conditions [13]. These results led to the selection of paclobutrazol as an effective treatment option in the present study to investigate its effects on the molecular mechanisms of rapeseed in more detail.

In winter rapeseed (Brassica napus L.), salt tolerance is achieved through various molecular mechanisms. These include the accumulation of osmolytes such as proline and glycine betaine, the activation of antioxidant enzyme systems like superoxide dismutase, catalase, and peroxidase, and the upregulation of genes involved in ion transport, such as NHX and SOS1. Among these mechanisms, flavonoids, particularly anthocyanins, play a crucial role in stress adaptation as secondary metabolites. Their strong antioxidant activity helps neutralize reactive oxygen species (ROS) generated under salt stress, thereby preventing oxidative damage to cellular macromolecules. Research has shown that salinity conditions lead to increased levels of anthocyanin accumulation in canola leaves and stems, which directly correlates with improved plant tolerance by enhancing the degradation of hydrogen peroxide and protecting cell membranes. Thus, anthocyanins serve not only as pigments but also as signaling and protective molecules within the intricate network of salt stress responses in canola [14–18].Flavonoids are crucial plant secondary metabolites that significantly contribute to cardiovascular health and cell immunity through their antioxidant, anti-inflammatory, and anticancer properties [19–21]. These compounds are generally synthesized in plants in two main steps, and their synthesis process is influenced by numerous genes and enzymatic factors. The initial step of this pathway begins with the production of precursors such as phenylalanine through the shikimic acid metabolic pathway [22–24]. In the subsequent steps, flavonoid synthesis is carried out through the activity of specific enzymes such as chalcone synthase (CHS). Enzymes such as cinnamate and picumarate also play a vital role in this process [25]. MYB111 and MYB12 genes are among the genes related to flavonoid synthesis, which are involved in flavonoid biosynthesis by binding to the promoter regions of key enzymes such as chalcone synthase (CHS) and flavonol synthase 1 (FLS1). These genes are activated under salt stress conditions and play an effective role in coping with this type of stress. In addition, MYB111 and MYB12 genes, together with other genes such as MYB12, TT4, TT5, and TT6, form a complex that is effective in regulating the synthesis and activation of flavonoids [26, 27]. ​​Given the significant role of these genes in flavonoid synthesis and their function in reducing the effects of salt stress, a detailed examination of these genes has been considered in the upcoming research. Finally, the aim of this study is to evaluate the effect of paclobutrazol treatment on the expression of MYB111 and MYB12 genes in rapeseed under salt stress conditions.

## Materials and methods

### Plant materials, growth conditions, and stress

In this study, seeds of the SLM046 cultivar were sourced from the Oilseeds Cultivation Development Company. The seeds were initially sterilized in a 3% sodium hypochlorite solution for one minute. After disinfection, they were rinsed thoroughly with autoclaved distilled water to ensure cleanliness. Subsequently, the seeds were cultured in glass jars and irrigated with autoclaved water. Throughout the growth period, which lasted 25 days, the glass jars were maintained under sterile and controlled conditions, including a temperature of 25 °C, with a light cycle of 16 h of light and 8 h of darkness in the growth room of the Biotechnology Research Institute. Regular irrigation with autoclaved water continued until the plants developed 2 to 3 leaves. Once this growth stage was reached, salinity stress was applied, and the final irrigation utilized a solution containing NaCl at a concentration of 150 mM.

### Cultivation conditions

Initially, seeds of the winter canola (*Brassica napus* L.) cultivar SLM046 were immersed in 70% ethanol for one minute. Subsequently, they were rinsed multiple times with autoclaved distilled water. Following this, the seeds were subjected to a 3% sodium hypochlorite solution for one minute and again thoroughly rinsed several times with autoclaved distilled water, thereby completing the seed sterilization protocol. The sterilized seeds were placed on sterile filter paper within glass jars and irrigated with autoclaved water to ensure complete saturation of the filter paper. The jars were maintained under strictly aseptic conditions in a growth chamber at the Biotechnology Research Institute, set at a temperature of 25 °C with a photoperiod of 16 h of light and 8 h of darkness. Regular irrigation with autoclaved water was conducted for a period of 25 days until the plants reached the growth stage of 2 to 3 true leaves, at which point the salinity stress treatment was initiated. This was performed by administering the final irrigation with a 150 mM NaCl solution. All harvested samples were immediately flash-frozen in liquid nitrogen and transferred to an ultra-low temperature freezer at −80 °C for long-term storage.

### Experimental and treatment design

Paclobutrazol is a biosynthetic gyberlin regulator that plays a significant role in regulating physiological processes, especially under drought stress conditions. In this research, 100 ppm of paclobutrazol was first prepared in distilled water, and then, according to the concentration of treatments, an appropriate volume was added to the environment. This study was carried out in a completely randomized design with three replications, and a total of 12 samples were examined. Of these, three samples were considered as controls and nine other samples as treatment groups. The control samples did not receive any paclobutrazol treatment. However, in the group of samples that were considered as treatments, the first three samples were affected by paclobutrazol treatment with a concentration of 10 PPM, the second three samples were treated with a concentration of 20 PPM paclobutrazol, and finally, the remaining three samples were treated with a concentration of 40 PPM paclobutrazol. In this research, the analysis of variance for the data was performed using the SAS software, and mean comparisons were calculated based on the Duncan test at the 5% significance level.

### Measurement of physiological parameters

To measure chlorophyll and carotenoids in rapeseed, the first step was to separate the leaves from the other vegetative parts of the plant. Next, 10 g of leaves were thoroughly ground in a porcelain mortar using a mixture of 16 ml of acetone and 4 ml of water. The resulting solution was then centrifuged at 2700 rpm for 10 min. After centrifugation, light absorption was measured at wavelengths of 663, 646, and 470 nm using a spectrophotometer. The amounts of chlorophyll a, chlorophyll b, and carotenoids were calculated using the following formulas [28].

$$\text{Chlorophyll a}=\left(12.25\times \text A663-2.79 \times \text A646\right)$$

$$\text{Chlorophyll b}=\left(21.21\times \text A646-5.1 \times \text A663\right)$$

$$\mathrm{Carotenoid}=\left(1000\times \text A470-1/8 \times \text {chl a}-85.02\times \text{chl b}\right)/198$$

### Expression study and real-time PCR analysis

After salt stress, leaves from both paclobutrazol-treated plants and control samples were collected. The samples were rapidly placed in liquid nitrogen and stored in a −80 °C freezer. Next, RNA extraction was conducted using the DENAzist kit, followed by cDNA synthesis with the appropriate Fermentas kit. Four primers was designed by Primer premier software (Table 1). The primers was checked by Primer Blast software. Finally, a real-time PCR reaction was carried out using the KTG Holdings proprietary kit and a Bioer device manufactured in China, employing primers for the Elongation Factor 1 gene as an internal control.

**Table 1:** Primers designed from MYB111and MYB12

	**Primer**	**TM**	**Product Size**
MYB111-F	CTCAGCCGCAAAATCTACGC	60°C	198 bp
MYB111-R	GGCCGTCAACATTTGCTTGT	60°C	198 bp
MYB12-F	CCGGGAGAACAGACAACGAA	60°C	178 bp
MYB12-R	TTGGGTTTCATGGCCGATCT	59.7°C	178 bp

### Molecular and bioinformatics analyses

In this study, we first conducted a detailed analysis of the MYB gene family, which is crucial for flavonoid biosynthesis. We focused specifically on the MYB111 and MYB12 genes due to their effectiveness and common usage in this process. Structural and molecular analyses of these genes were performed using the Expasy database, which included measurements of isoelectric points, calculations of molecular weights, determination of GC percentages, and precise gene localization. The results of these analyses are summarized in Table 2. Next, we examined the three-dimensional structures and stability of the proteins using the Swiss Model database, and we conducted protein network analysis with the String database. Additionally, we performed a phylogenetic analysis of the protein sequences using MEGA5 software. To assess the similarity of these protein sequences with others, we carried out a protein BLAST analysis using the Circoletto database. Finally, we conducted a hydropathicity analysis using the ProtScale database and examined the secondary structures of the MYB111 and MYB12 proteins using the Proteus database.

**Table 2:** Molecular identification and analysis of MYB111 and MYB12 genes

Name	*MYB111*	*MYB12*
ORGANISM	*Brassica napus*	*Brassica napus*
Accession number nucleotide	XM_013803650.3	XM_013892082.3
Accession number protein	XP_013659104.2	XP_013747536.2
Gene symbol	LOC106364001	LOC106450432
Chromosome	A9	C4
Location	2,656,760..2659586	258,022..261701
GeneID	106,364,001	106,450,432
nucleotide length	1554 bp	1803 bp
protein length	349 aa	390 aa
Molecular weight (Da)	39,383.54	43,618.15
Isoelectric point	5.24	5.27
Exon	3	3
GC	40.7%	43.3%

## Results

### Analysis of physiological indices

**Fig. 1:**
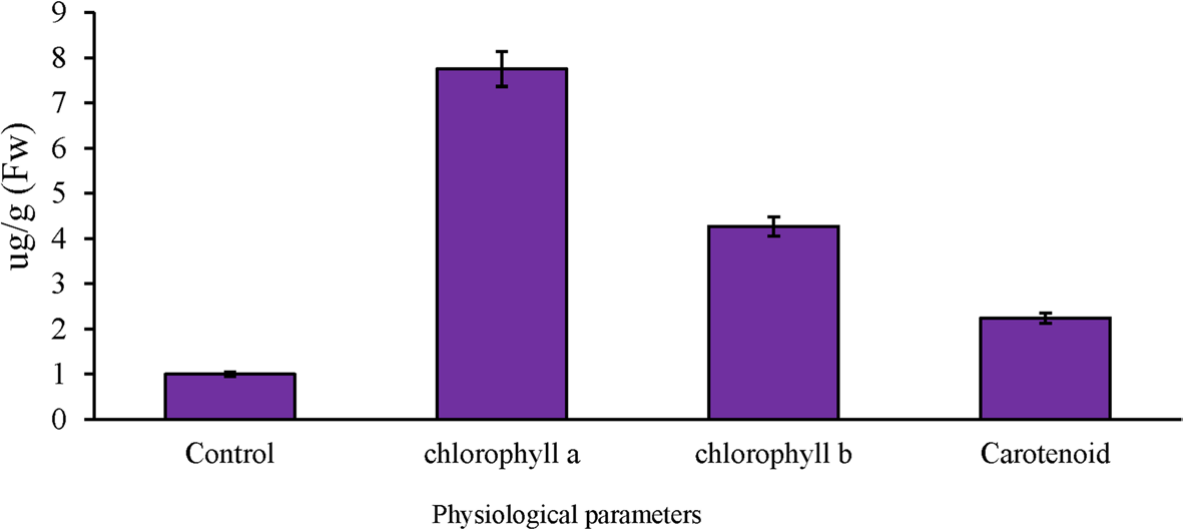
Analysis of physiological indices in *Brassica napus* leaves under the influence of paclobutrazol treatment

**Fig. 2:**
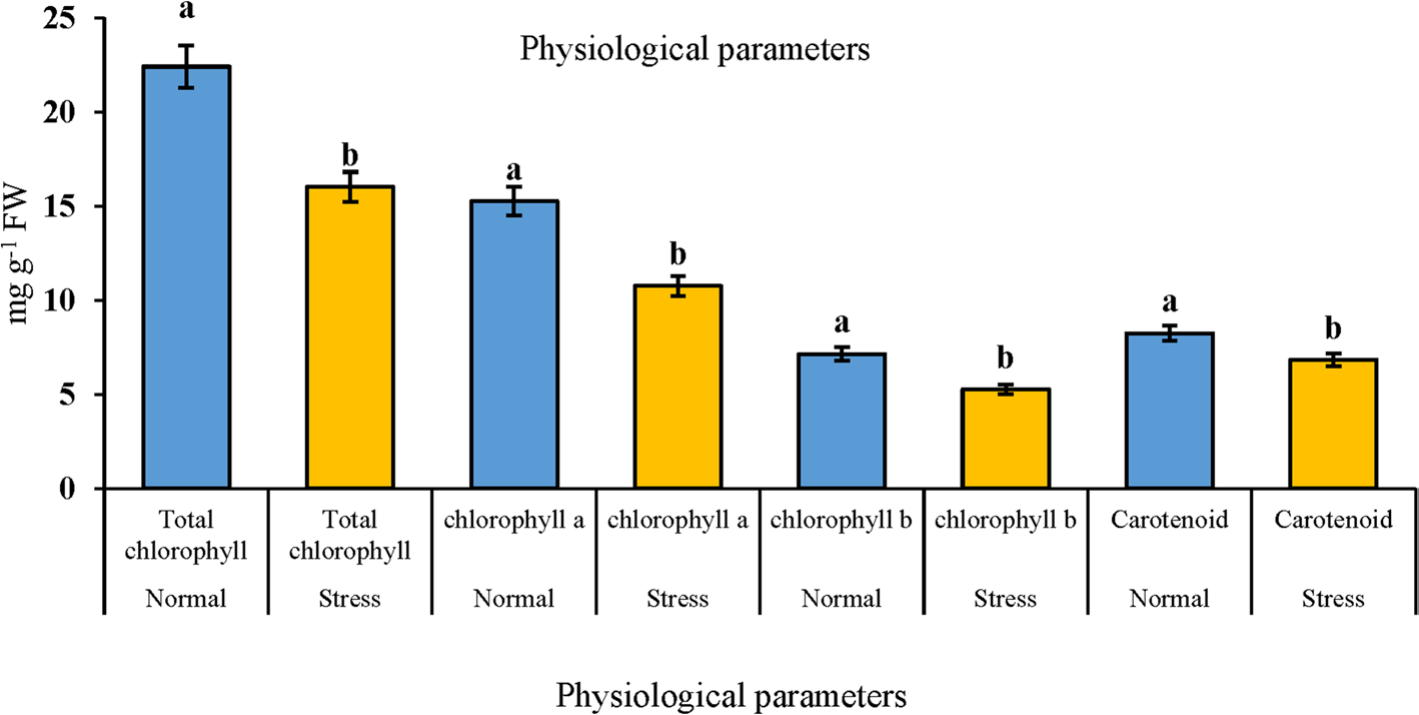
Determination of some physiological parameters

In this study, we measured the levels of chlorophyll a, chlorophyll b, and carotenoids in both control and treated leaves exposed to a concentration of 40 PPM paclobutrazol, using a spectrophotometer. The results, illustrated in Fig. 1, indicate that the treated plants contain higher levels of chlorophyll and carotenoids compared to the control plants. This significant difference confirms that paclobutrazol treatment positively enhances the physiological indices of the plants. Additionally, we found that chlorophyll and carotenoid levels decreased under salt stress conditions compared to the control. Concurrently, physiological analyses revealed significant increases in malondialdehyde (MDA), superoxide dismutase (SOD), peroxidase (POD), and electrolyte leakage (EL) under salt stress compared to the control. These analyses were conducted at the stages of stem formation, flowering, and grain filling, as shown in Fig. 2.

#### Determination of MDA content

**Fig. 3:**
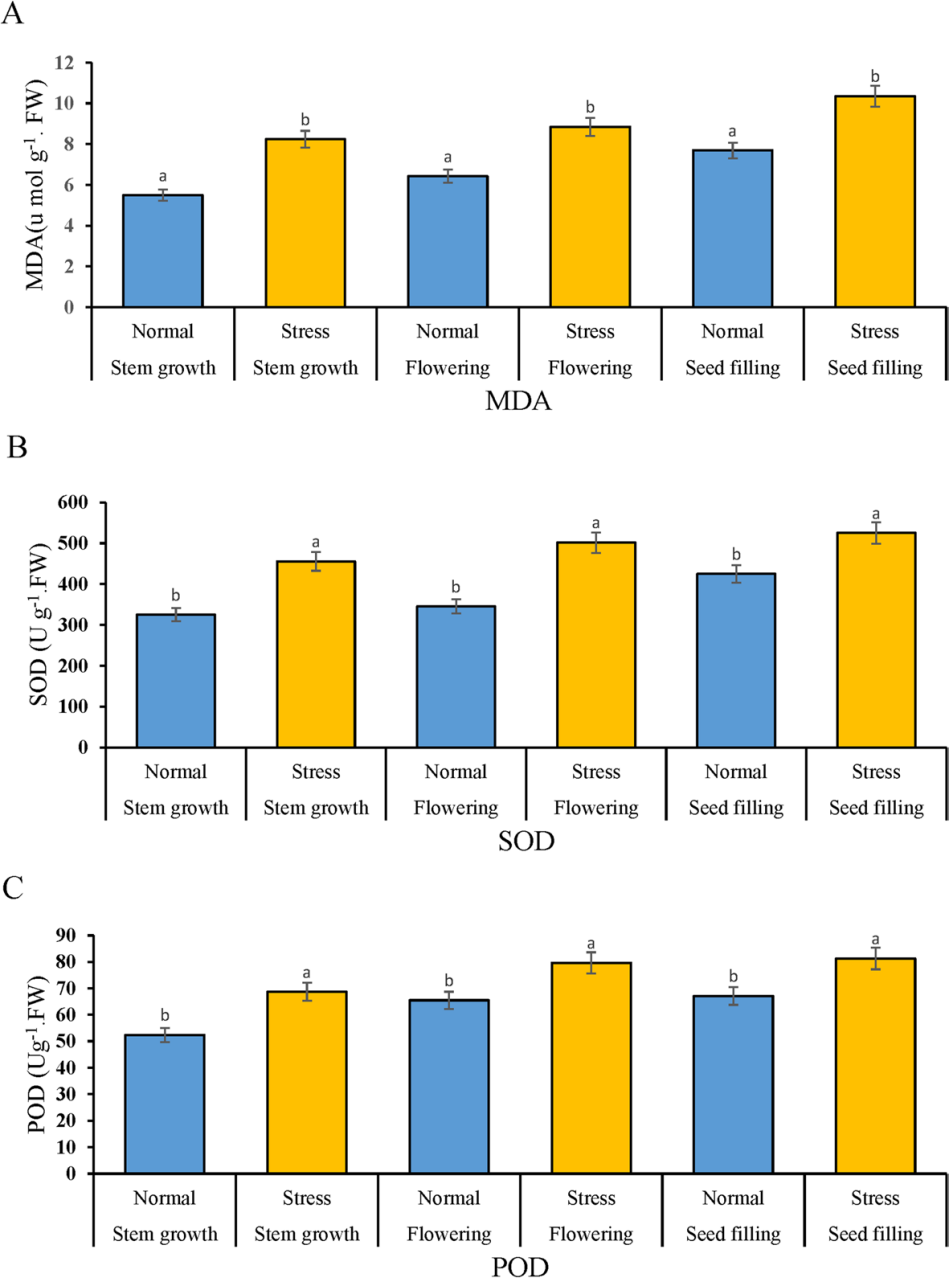
Determination of (**A**) MDA, (**B**) SOD, and (**C**) POD content

The generation of reactive oxygen species (ROS) under salinity stress induces lipid peroxidation of the cell membrane, leading to the formation of intermediate compounds such as short-chain hydrocarbons, ketones, and malondialdehyde (MDA), with MDA being the most significant. These intermediates can react with thiobarbituric acid (TBA) to produce a chromogenic compound, collectively termed thiobarbituric acid reactive substances (TBARS), which serve as an estimate of the extent of membrane lipid peroxidation. Membrane lipid peroxidation represents the most deleterious impact of oxygen free radicals on plants under stress conditions, ultimately culminating in cell death. Quantifying the degree of membrane lipid peroxidation alone can be employed as a reliable indicator of cellular damage. The extent of membrane lipid peroxidation reflects the severity of stress imposed on the plant, and malondialdehyde concentration can be utilized as a biochemical index to assess membrane stability and the magnitude of membrane injury (Fig. 3A).

#### Determination of SOD content

Superoxide dismutase (SOD) is one of the most powerful intracellular antioxidants and is often considered the strongest known antioxidant. It protects many plants from oxidative damage caused by oxygen free radicals and enhances their resilience to various environmental stressors. Researchers have identified multiple isozymic forms of this enzyme in plants, which are categorized into three groups based on their metallic cofactors: Mn-SOD is found in mitochondria and peroxisomes, Cu/Zn-SOD is located in chloroplasts and the cytosol, and Fe-SOD is present in chloroplasts. SOD catalyzes the dismutation of two superoxide molecules through a concerted reaction involving protonation, converting them into two molecules of hydrogen peroxide. This hydrogen peroxide is then scavenged by other antioxidant systems. Additionally, by keeping superoxide concentrations low, SOD helps minimize the formation of hydroxyl radicals. This study revealed that salinity stress in rapeseed significantly increases superoxide dismutase activity, highlighting the critical role of this enzyme in detoxifying oxygen free radicals (Fig. 3B).

#### Determination of POD content

Another critical antioxidant is peroxidase (POD), which exists in diverse isoforms and is responsible for scavenging hydrogen peroxide from biological systems. Owing to its capacity to oxidize guaiacol, it is also termed guaiacol peroxidase (GPOX). Peroxidases are recognized for their distinct physiological roles compared to other antioxidants (Fig. 3C).

#### Relative electrolyte leakage (EL)

**Fig. 4:**
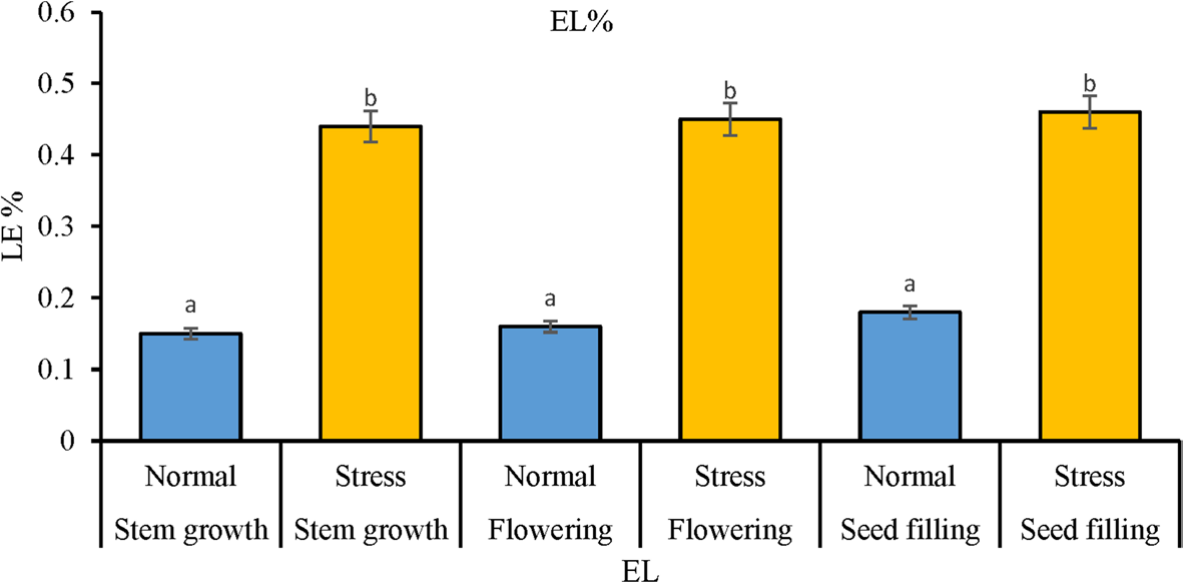
Determination of EL percent

This index was measured according to the Sairam method. According to this method, 0.1 g of leaf is placed in 10 ml of double distilled water. After that, the sample is placed in 40° C water for 30 min and its electrical conductivity (EC) is read with the help of an EC meter. (C1) Then, the sample is placed in a bain-marie bath at 100° C for 15 min and its electrical conductivity is read for the second time (C2). And the desired indices are calculated based on the following relations (Fig. 4).

$$\mathrm{EL}=\text C_1/ \text C_2$$

### Analysis of MYB111 and MYB12 gene expression profiles

**Fig. 5:**
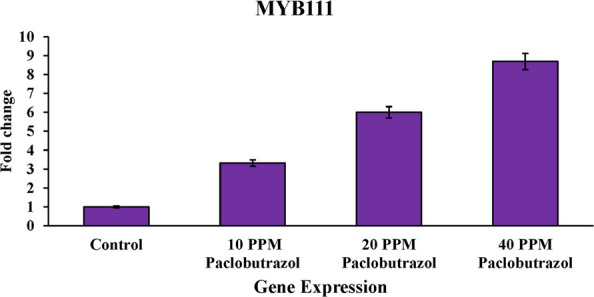
Analysis of MYB111 gene expression under the influence of different concentrations of paclobutrazol treatment

**Fig. 6:**
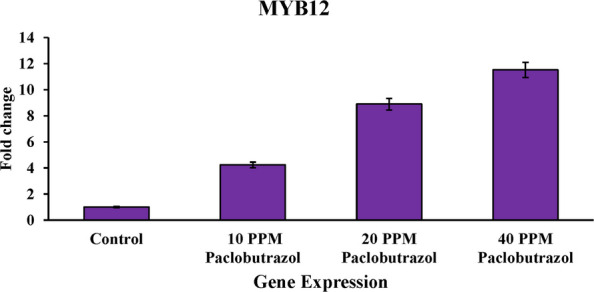
Analysis of MYB12 gene expression under the influence of different concentrations of paclobutrazol treatment

In this study, rapeseed cultivar SLM046, which is relatively tolerant to salinity, was used. In this research, we tried to investigate the effects of paclobutrazol treatment on the changes in the expression profile of MYB111 and MYB12 genes under salt stress. For this reason, RNA extraction and cDNA synthesis were done from the stressed and control (no paclobutrazol treatment) samples, and then the samples were placed in the real-time PCR machine to check the actual expression of the genes under salt stress. The results showed that the paclobutrazol treatment increased the expression of MYB111 and MYB12 genes compared to the control (no paclobutrazol treatment) state. It was also found that with increasing treatment concentration, the expression level of these genes also increased. Comparison of the expression profiles showed that MYB12 gene expression under the influence of paclobutrazol is at a higher level than MYB111. Changes in the expression levels of MYB111 and MYB12 genes are clearly shown in Figs. 5 and 6 under the influence of different treatment concentrations.

### Network analysis and determination of the three-dimensional structure and stability of MYB111 and MYB12 proteins

**Fig. 7:**
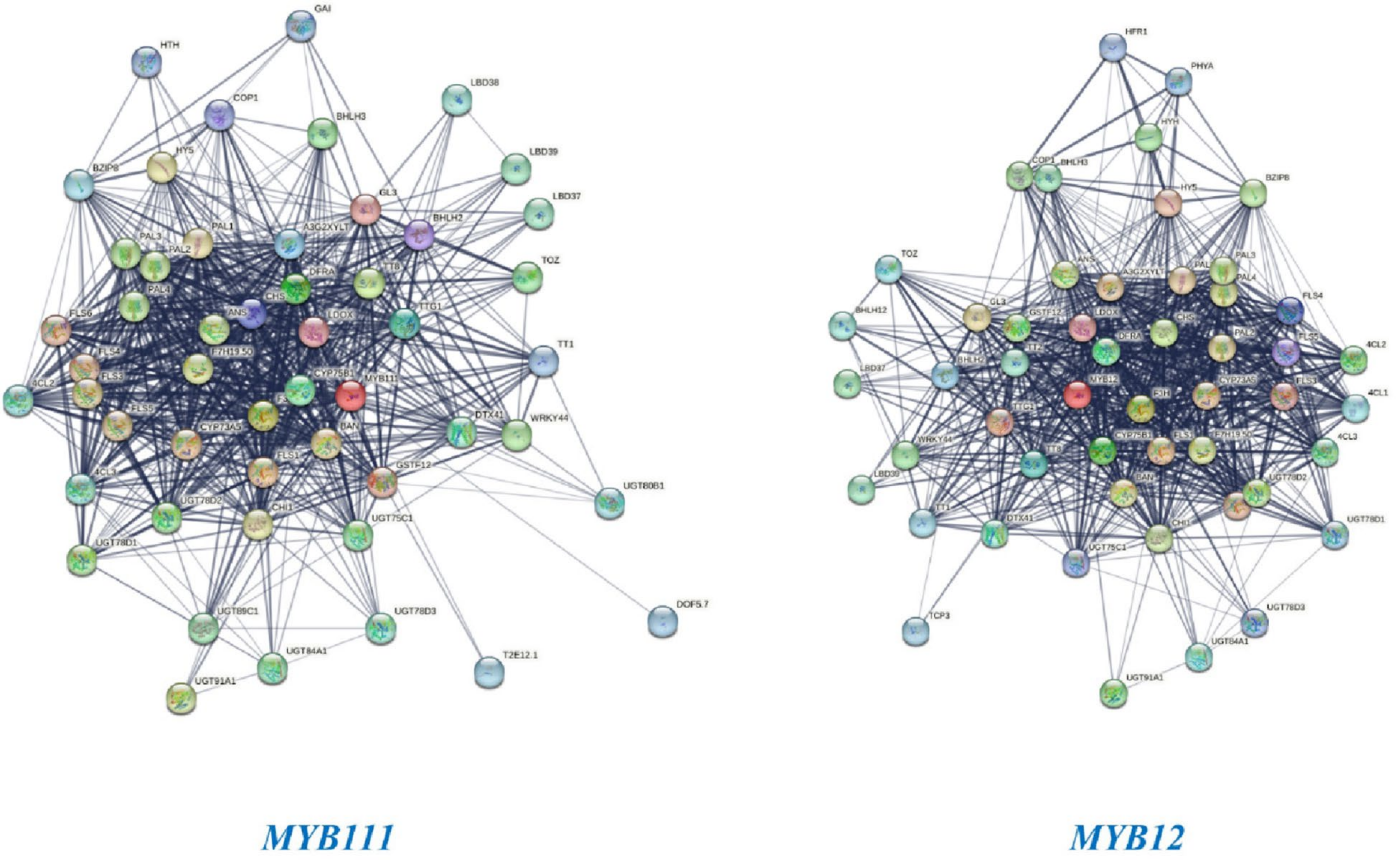
Results from network analysis of MYB111 and MYB12 genes. Red circles indicate the target genes MYB111 and MYB12, while other circles represent 50 genes related to these genes

**Fig. 8:**
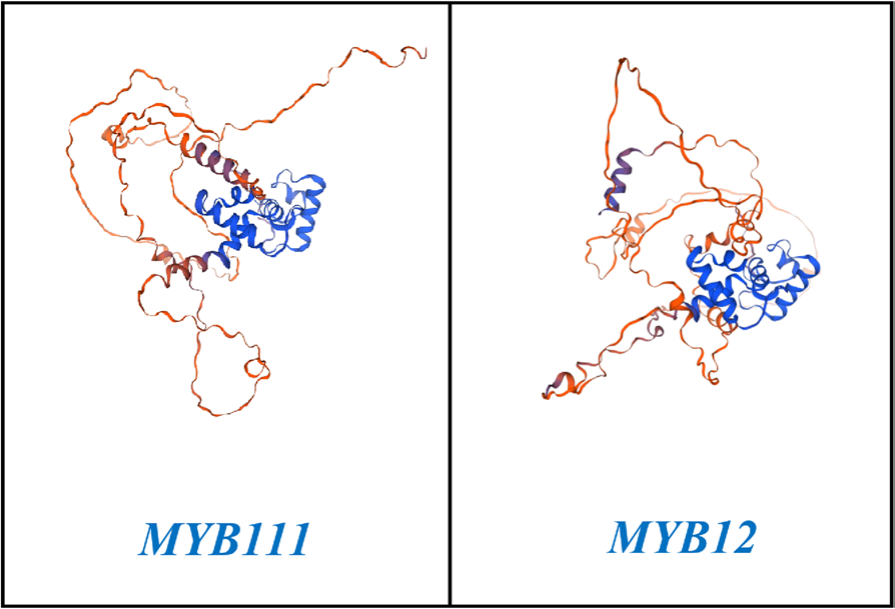
Three-dimensional structure of MYB111 and MYB12 proteins

**Fig. 9:**
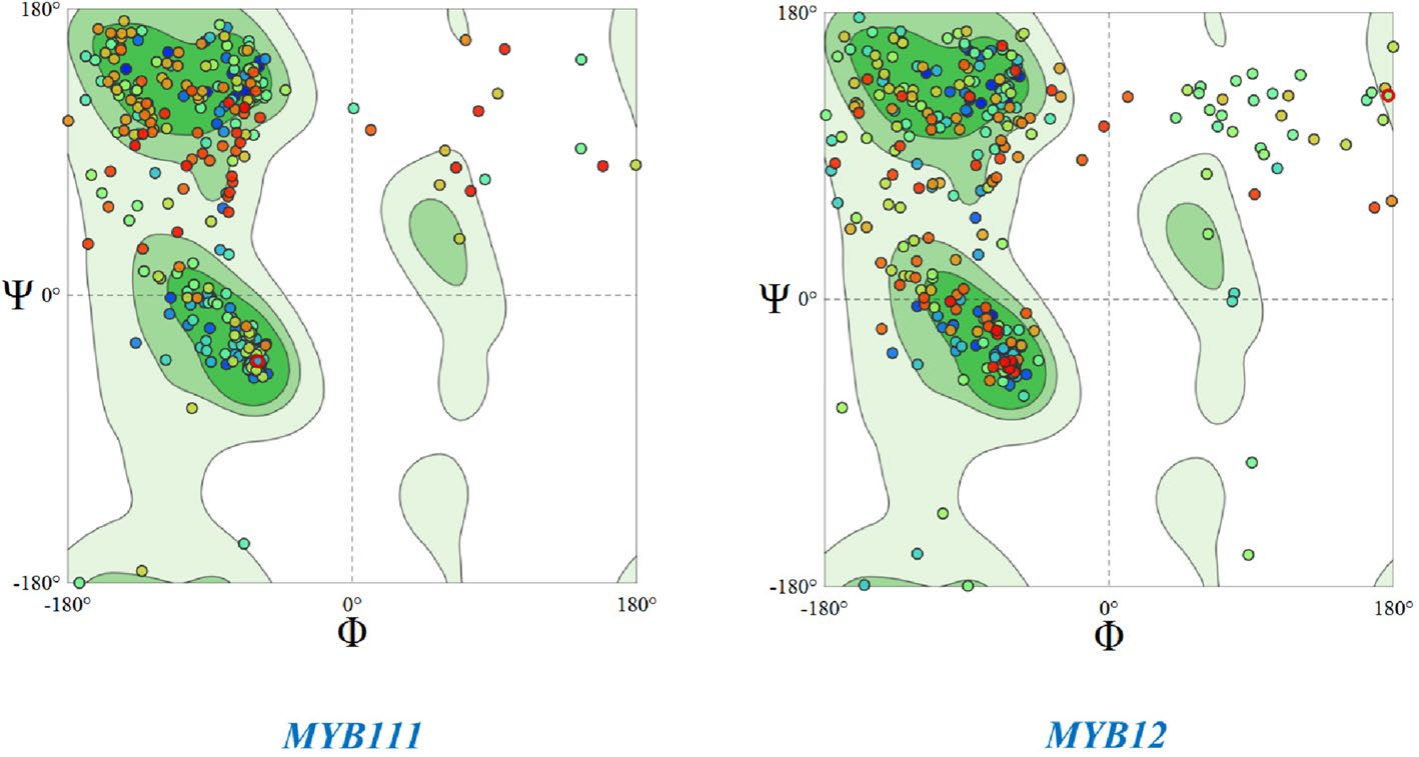
Ramachandran plot of MYB111 and MYB12 proteins

Network analysis of the MYB111 and MYB12 genes using the STRING database has shown that these genes interact with a wide network of genes and transcription factors. This communication network generally leads to the production of antioxidants and increased plant resistance to stresses such as salinity and drought. Figure 7 depicts the network structure of the MYB111 and MYB12 genes. The three-dimensional structure and stability of the MYB111 and MYB12 proteins were also examined using the Swiss Model database. The results of the three-dimensional structure analysis of the MYB111 protein in rapeseed showed that this structure is 81.63% similar to that of its similar protein in Arabidopsis thaliana. In addition, the three-dimensional structure of the MYB12 protein in rapeseed also showed 79.11% similarity to the MYB12 protein in Arabidopsis thaliana (Fig. 8). Also, the Ramachandran plot was calculated for the MYB111 and MYB12 proteins with respect to the φ and ψ angles. This analysis showed that the stability and energy levels of these proteins are 85.04% and 73.45%, respectively. Based on these values, it can be concluded that both proteins are at an acceptable level of stability, but the MYB111 protein is more stable than the MYB12 protein. Figure 9 shows the Ramachandran plot in terms of the φ and ψ angles in the MYB111 and MYB12 proteins.

### BLAST analysis and phylogenetic tree study of MYB111 and MYB12 proteins

**Fig. 10:**
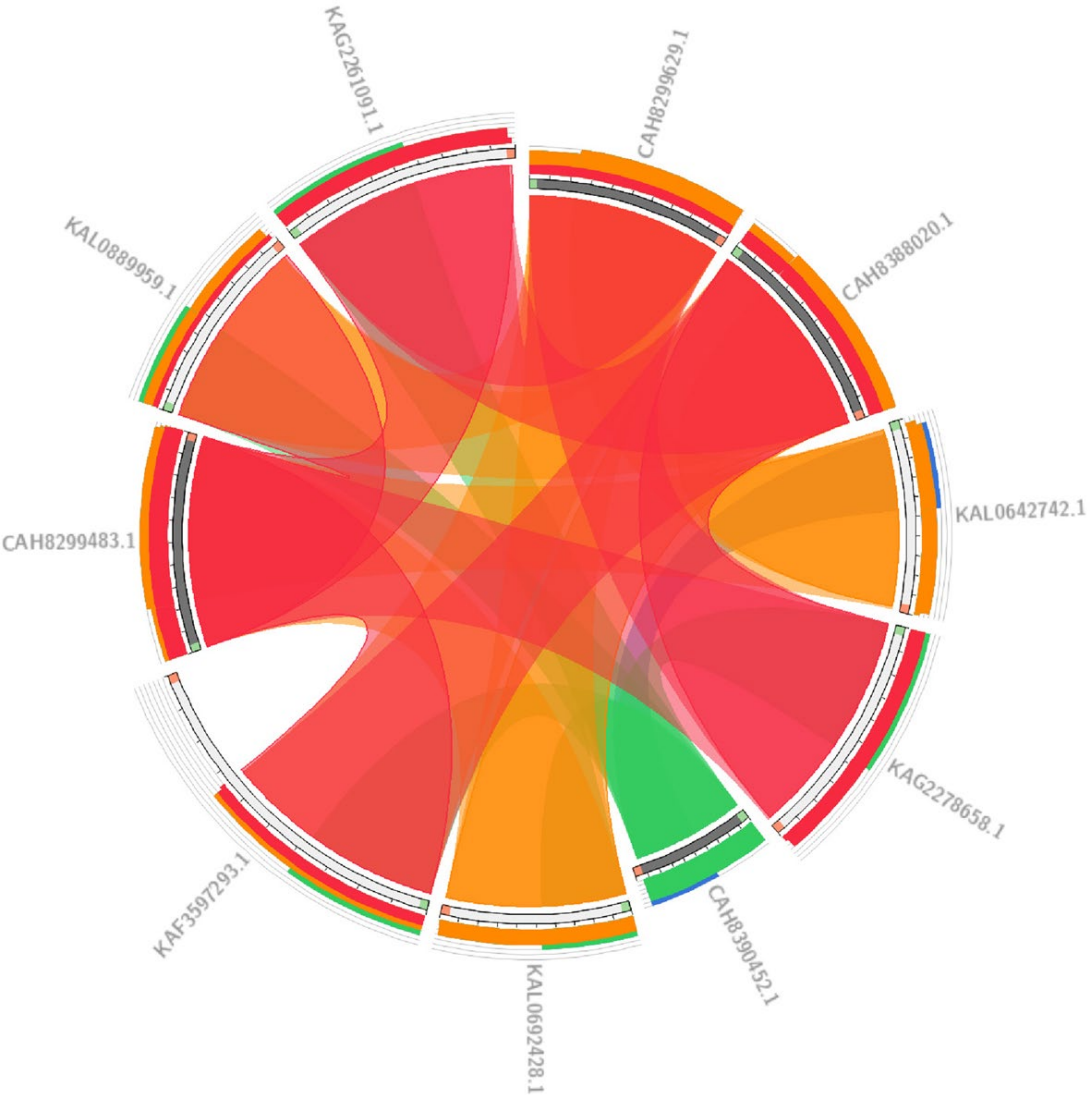
Blast analysis of MYB111 and MYB12-related proteins in *Brassica carinata* and *Eruca vesicaria*. Blue bands indicate less than 25% similarity, green bands indicate less than 50% similarity, orange bands indicate less than 75% similarity, and red bands indicate more than 75% similarity

**Fig. 11:**
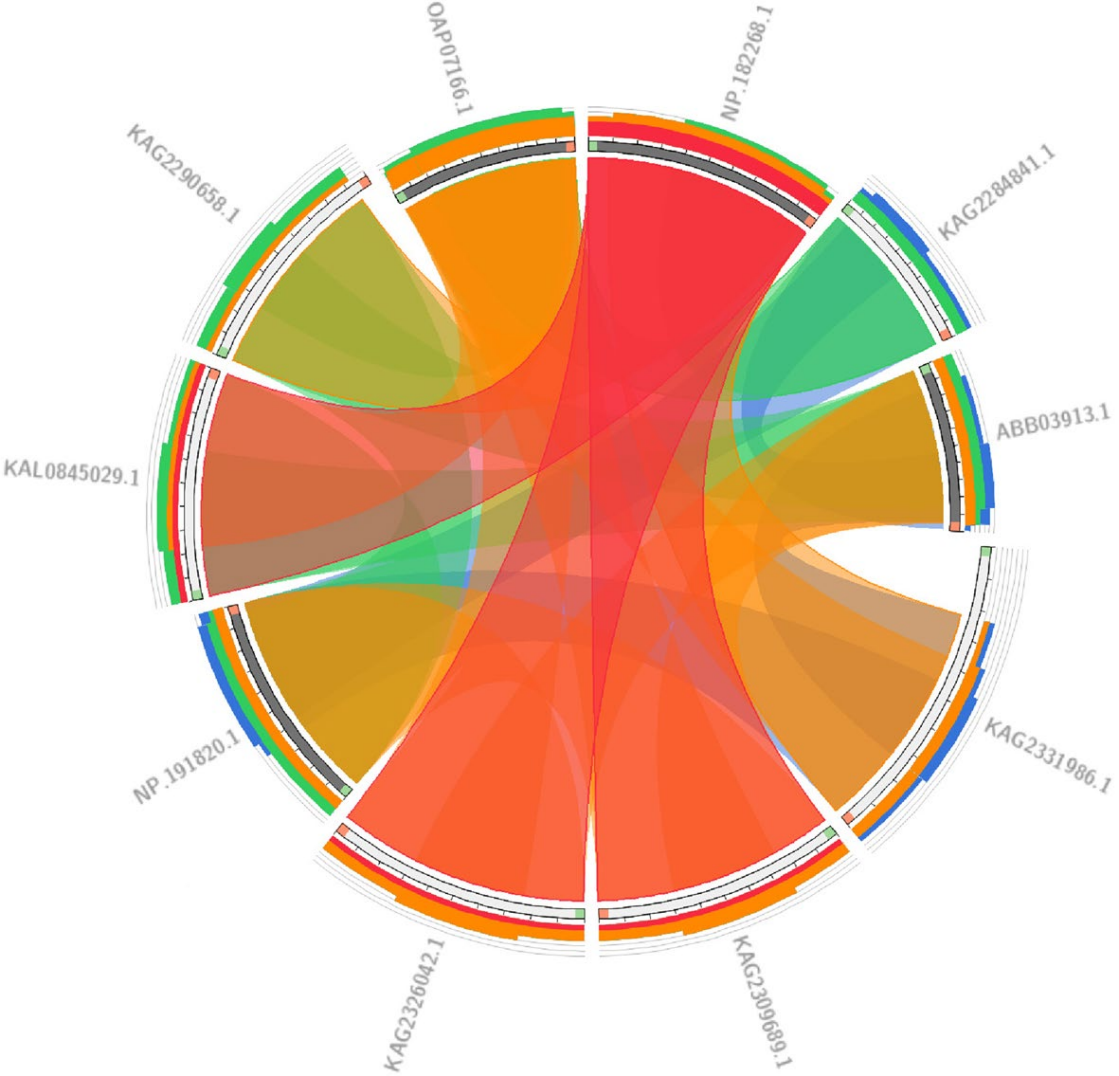
Blast analysis of MYB111 and MYB12-related proteins in *Arabidopsis thaliana* and *Brassica carinata*. Blue bands indicate less than 25% similarity, green bands indicate less than 50% similarity, orange bands indicate less than 75% similarity, and red bands indicate more than 75% similarity

**Fig. 12:**
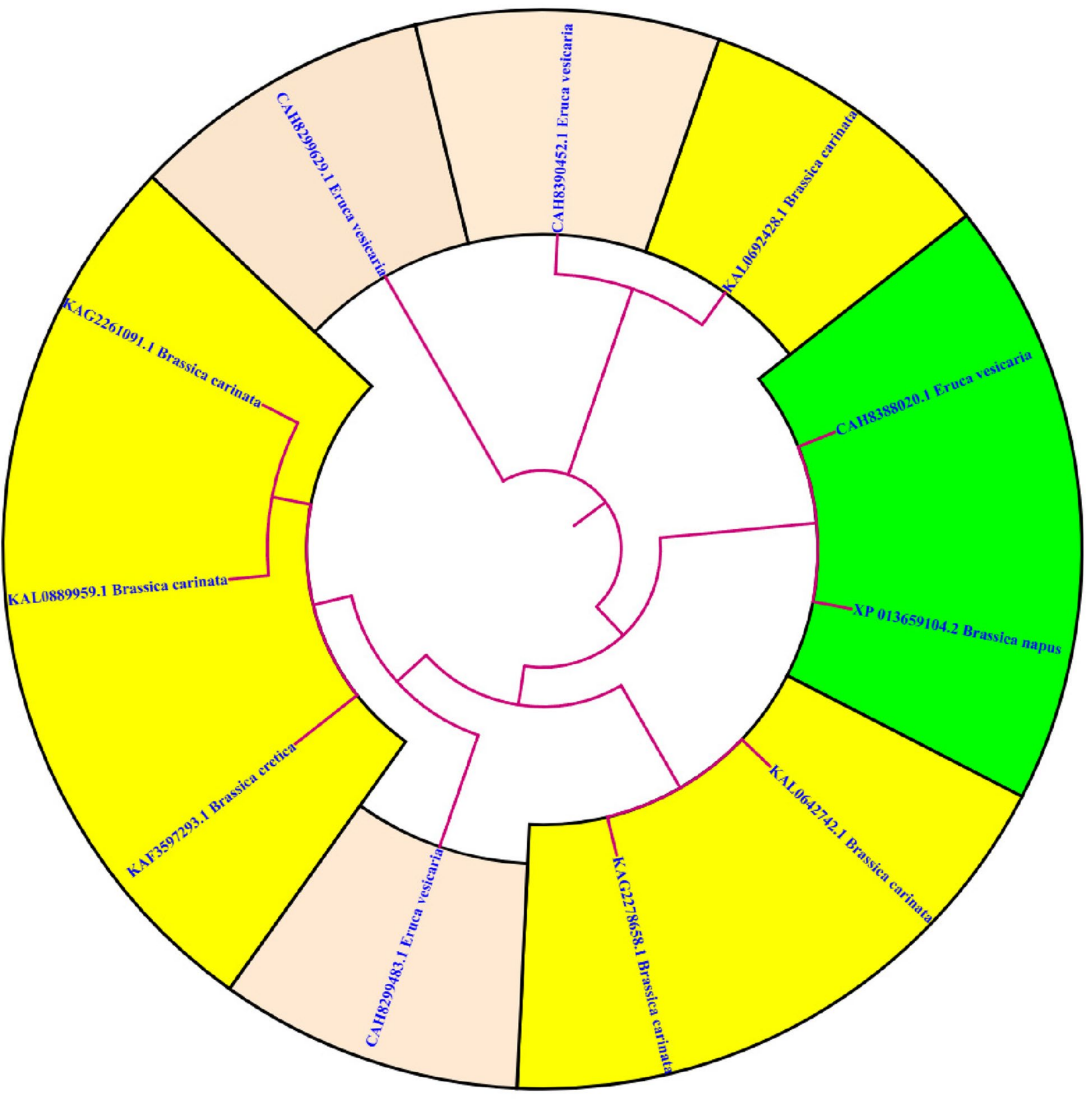
Phylogenetic tree analysis of MYB111-related proteins in *Brassica carinata* and *Eruca vesicaria*. As can be seen, the sequence CAH8388020.1 (*Eruca vesicaria*) has the highest similarity to the (XP_013659104.2) protein

**Fig. 13:**
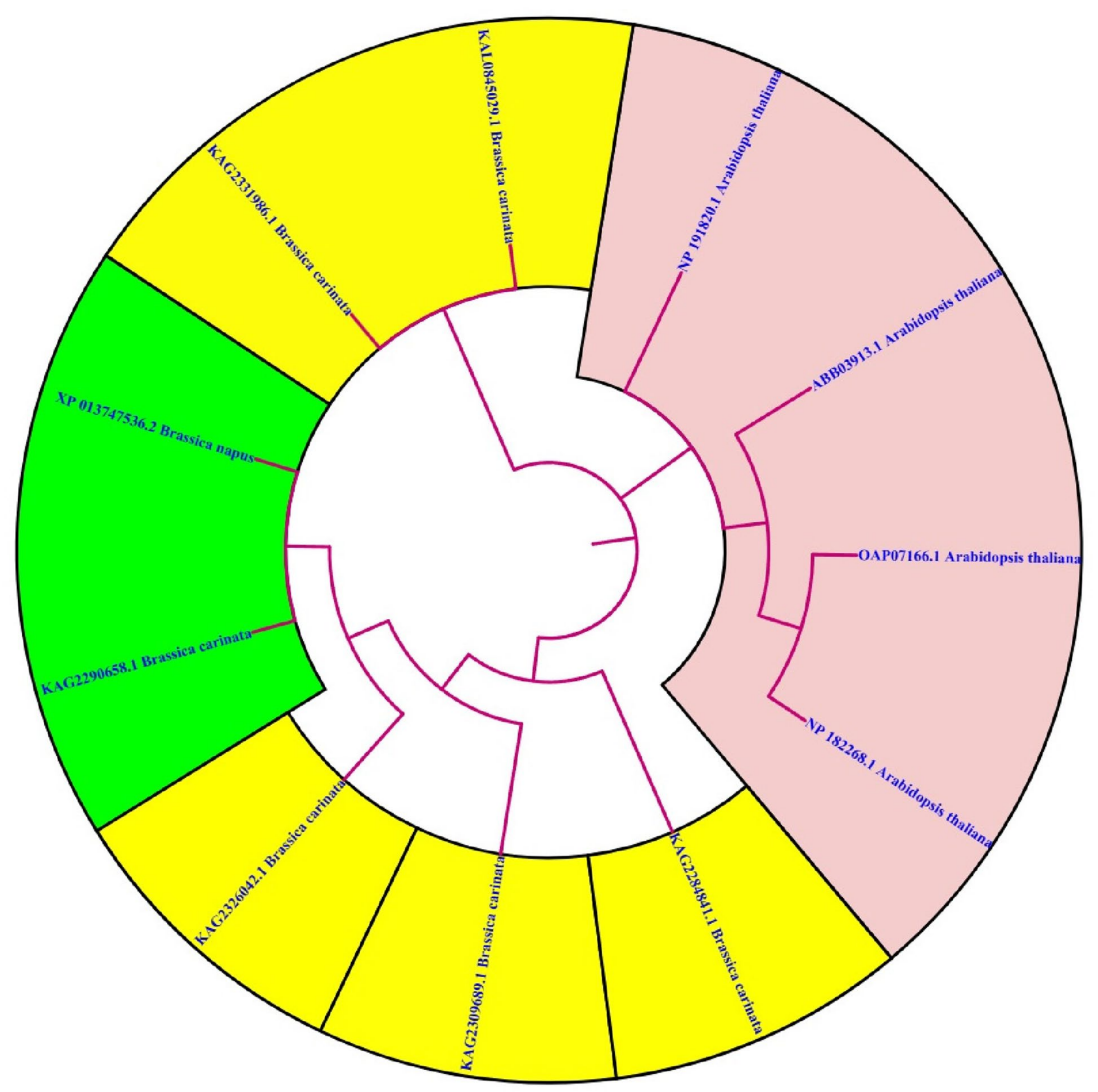
Phylogenetic tree analysis of MYB12-related proteins in Arabidopsis thaliana and Brassica carinata. As can be seen, the sequence KAG2290658.1 (*Brassica carinata*) has the highest similarity to the (XP_013747536.2) protein

BLAST analysis, both at the nucleotide and protein levels, is widely used for identifying and comparing sequences across different species. Such studies can lead to the discovery of previously unknown genes and proteins in various organisms. In this study, the Circoletto database was used to compare protein sequences. First, the protein sequences of *Brassica carinata* and *Eruca vesicaria* species that had more than 50% similarity to the MYB111 protein sequence in *Brassica napus* were extracted from the NCBI database. Then, BLAST analysis was performed using the Circoletto database on the protein sequences. The results of BLAST analysis showed that a total of 24 protein bands were formed. These bands showed a minimum similarity of 53.85% and a maximum similarity of 88.05%. Also, for the MYB12 protein sequence, proteins with more than 50% similarity to Brassica napus were extracted from *Arabidopsis thaliana* and *Brassica carinata* species, which ultimately led to the formation of 24 bands with a minimum similarity of 32.91% and a maximum similarity of 77.08%. Figures 10 and 11 present the results of the BLAST analysis for the MYB111 and MYB12 protein sequences, respectively. Phylogenetic tree analysis is a valuable tool in bioinformatics that classifies sequences based on their similarity, enabling researchers to analyze them more effectively. The phylogenetic tree analyses for the MYB111 and MYB12 protein sequences are illustrated in Figs. 12 and 13, respectively.

### Secondary structure and hydropathicity analysis of the MYB111 and MYB12 proteins

**Fig. 14:**
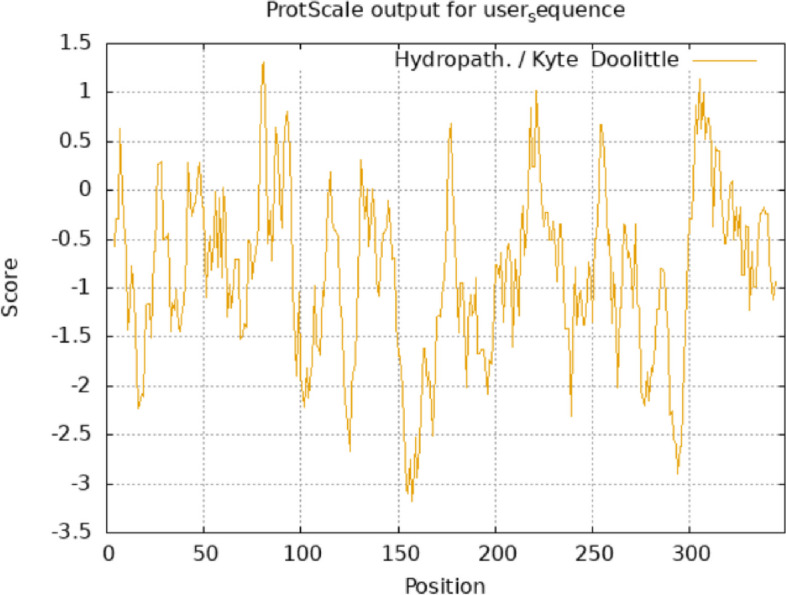
Hydropathicity diagram of MYB111 protein. The average, minimum, and maximum hydropathicity of MYB111 protein are equal to AV = −0.915772201, MIN = −3.178, and MAX = 1.311

**Fig. 15:**
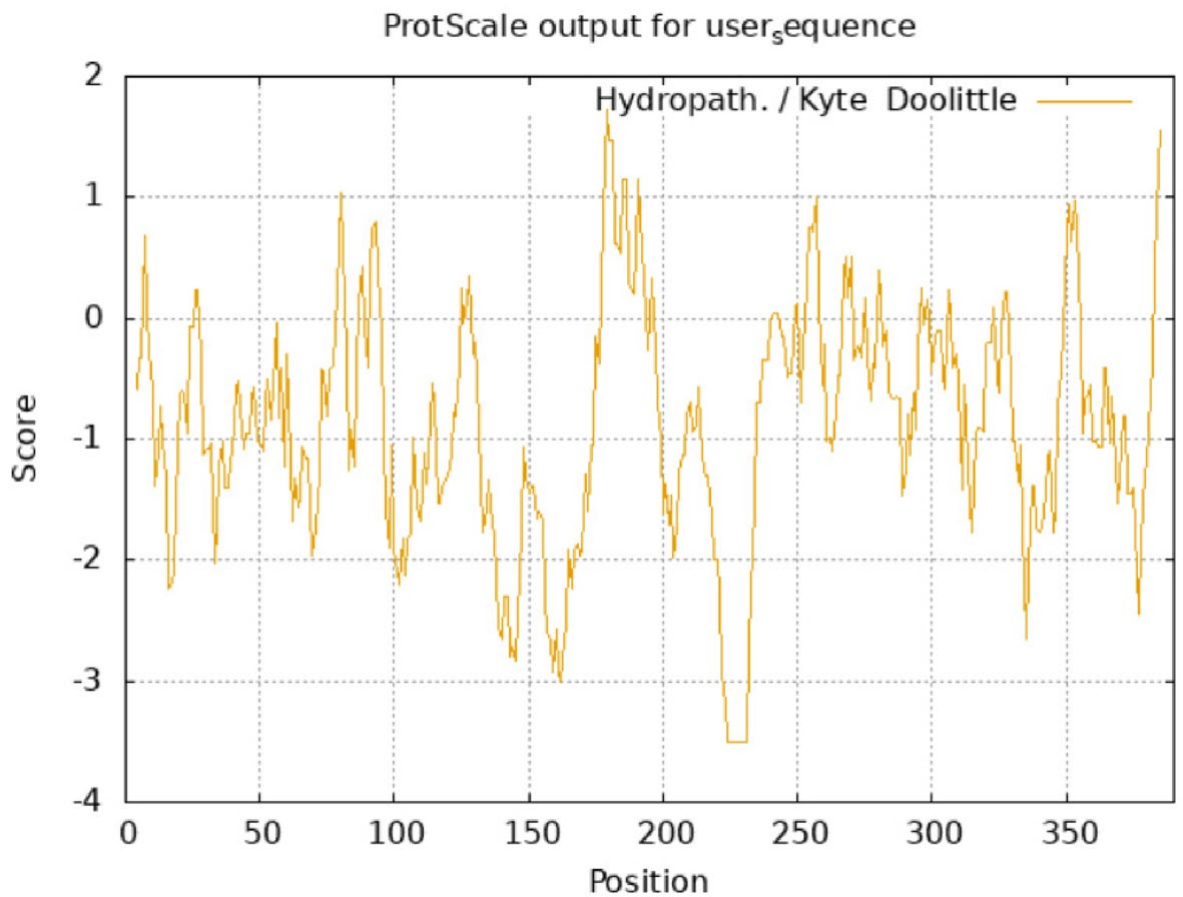
Hydropathicity diagram of MYB12 protein. The average, minimum, and maximum hydropathicity of MYB12 protein are equal to AV = −0.885126667, MIN = −3.500, and MAX = 1.722

The analysis of protein secondary structure provides valuable insights into the spatial arrangement and folding of peptides in specific regions. In this study, we determined the secondary structure of the MYB111 and MYB12 proteins using the PROTEUS2 database. The results of this analysis are presented in Table 2. Additionally, hydropathicity analysis revealed that both MYB111 and MYB12 proteins exhibit a negative average hydropathicity, classifying them as hydrophilic proteins. Figures 14 and 15 display the hydropathicity analysis results for MYB111 and MYB12 proteins, respectively (Table 3).

**Table 3:** Results of the secondary structure analysis for the proteins MYB111 and MYB12

Secondary structure analysis	MYB111	MYB12
Number of sequence alignments used for ab-initio predictions	49	49
Overall confidence value	74.8%	73.0%
Helix content	20%	24%
Beta sheet content	0%	2%
Coil content	80%	74%
Signal peptide content	0%	0%
membrane content	0%	0%

## Discussion

In recent years, researchers have conducted extensive studies to understand the mechanisms of resistance to abiotic stresses, particularly salt stress. A clear understanding of these mechanisms can enhance plant resilience to saline conditions. Additionally, the use of compounds and regulatory factors can effectively mitigate the harmful effects of salinity. While research in biology and genetic engineering has shown that sensitivity to salt stress occurs upstream of certain genes, with flavonoid production occurring downstream, the complex interactions between these factors remain unclear. Therefore, this study aimed to investigate the changes in antioxidants and the genes involved in this process by utilizing paclobutrazol treatment, a significant growth regulator. Our findings demonstrate that paclobutrazol treatment interacts with transcription factors related to salt and drought stress, leading to changes in intracellular hormone levels [29, 30].

The results of this study demonstrated that paclobutrazol significantly increases the mineral content of rapeseed. Additionally, the gene expression analysis revealed that a concentration of 40 ppm of paclobutrazol enhances the expression of genes associated with flavonoid production. These findings suggest that paclobutrazol acts as a stimulant for flavonoid production, thereby increasing secondary metabolites in rapeseed. This aligns with the findings of Zhang et al. in 2025, who also reported that paclobutrazol stimulates antioxidant biosynthesis and increases levels of flavonoids, anthocyanins, and terpenoids [31].

While paclobutrazol (PBZ) is effective for controlling vegetative growth in fruit trees, it carries significant agronomic risks. Long-term use or improper dosing of this gibberellin biosynthesis inhibitor can result in excessive dwarfing, reduced leaf area, and diminished photosynthetic capacity. Additionally, since gibberellins play a crucial role in inducing flowering in certain species, inappropriate PBZ application may delay the transition to the reproductive stage, disrupt flower bud formation, and lead to uneven crop maturity. Other risks include disturbances to the plant's hormonal balance and the buildup of persistent residues in the soil, which can negatively impact subsequent crop cycles. To optimize PBZ use and mitigate side effects, it is essential to determine the correct dose based on factors such as plant species, tree age, and environmental conditions. A key strategy is to target applications at specific growth stages—such as early in the growing season to manage vegetative growth—while avoiding use during sensitive periods, like near flowering. The choice of application method (soil or foliar) should also align with the desired duration of effect and manageability. Overall, integrating PBZ with other growth management practices, such as pruning and regular soil monitoring to prevent residue buildup, is crucial for a balanced and sustainable management program [32–34].

Paclobutrazol activates the expression of the evolutionarily conserved transcription factors MYB111/12 by modulating intracellular hormonal balance. These transcription factors, in turn, increase flavonoid biosynthesis by binding to the promoters of key constitutive genes (e.g. CHS, F3H, FLS). This integrated interpretation provides a strong bridge between bioinformatics analyses and the physiological consequences of PBZ treatment [35–39]. To complete this picture, future studies could focus on directly verifying the binding of MYB111/12 proteins to the promoters of target genes under PBZ treatment conditions, as well as directly measuring hormone levels.

Studies show that PBZ acts as a potent antioxidant and a metabolic regulator and uses several mechanisms to cope with salinity. Among others, this compound reduces the level of destructive reactive oxygen species (ROS) by increasing the activity of antioxidant enzymes such as superoxide dismutase (SOD), catalase (CAT), and peroxidase (POD), preventing oxidative damage to membranes and macromolecules. PBZ also increases the accumulation of compatible osmolytes such as proline and glycine betaine, which contribute to the maintenance of cell osmotic potential and the integrity of protein structures. On the other hand, reports suggest that PBZ may selectively inhibit the uptake of sodium ions (Na⁺) and, in turn, facilitate the uptake of potassium ions (K⁺), leading to an improvement in the vital K⁺/Na⁺ ratio in plant tissues. These physiological and biochemical changes are ultimately evident in the form of significant improvements in growth indices such as plant height, leaf area, chlorophyll content, and dry weight in salt-stressed plants treated with PBZ (especially at optimal concentrations such as 100 mM) compared to stressed control plants. These findings strongly support the aforementioned proposition that PBZ plays a mitigating role on the negative effects of salt stress [40–43].

Bioinformatics analyses, particularly the evaluation of the MYB111 and MYB12 genes, revealed significant interactions with transcription factors and regulatory elements related to reactive oxygen species (ROS). These findings suggest that increased expression of these genes enhances the activity of ROS scavengers. As a result, elevated flavonoid production in plants not only boosts antioxidant activity but also reduces the damaging effects of ROS. In a related study, Li et al. (2019) demonstrated that the upregulation of MYB111 in Arabidopsis lowers ROS levels and improves the plant's tolerance to salinity. Their findings further confirm the crucial role of MYB111 in enhancing the plant's ability to withstand salt stress [44]. Bioinformatics analyses of this study also showed that flavonoid-synthesizing genes have negative hydropathicity. This means that these genes are associated with transcription factors that increase drought stress resistance. As a result, it can be said that MYB111 and MYB12 genes, in addition to providing resistance to salinity, also increase drought stress resistance. As the findings of Qi et al. in 2025 showed, the Astragalus membranaceus plant becomes more resistant to salinity and drought stress by increasing flavonoid production [45].

It has been investigated the accumulation of flavonoids and the reduction of reactive oxygen species production through MYB transcription factors in the Schima superba family, and their results indicated an increase in drought stress resistance in this family [46]. The results of this study, particularly the protein blast and phylogenetic analyses, indicate that the MYB111 and MYB12 genes are linked to R2R3-MYB transcriptional activators in both protoplasts and the nucleus. These activators promote genes associated with abscisic acid, enhancing plant resistance to abiotic stresses. Therefore, it can be concluded that flavonoid synthesis is crucial for improving plant resilience against such stresses.

Since previous studies have well proven the role of MYB111 and MYB12 genes in improving salt stress. In this study, when paclobutrazol treatment was used, the expression of these genes increased significantly. As a result, by combining the findings of this study and previous studies, it is concluded that paclobutrazol treatment has a positive effect on increasing salt resistance [36, 44, 47].

## Conclusion

Salinity, as a factor that leads to excessive accumulation of salts and destruction of soil structure, has significant negative effects on oilseed production. In rapeseed, this environmental stress directly disrupts the germination process and shoot growth, and also reduces the production of flavonoids and anthocyanins. This study showed that the use of paclobutrazol treatment can improve cellular activities under environmental stresses. In addition, the expression of flavonoid synthesis-related genes production is also significantly increased under the influence of paclobutrazol. These mechanisms can significantly reduce the negative effects of salt stress. Overall, the results of this study indicate that the use of paclobutrazol in oilseeds can reduce the accumulation of sodium ions in the plant structure.

## Data Availability

All the data are embedded in the manuscript. The datasets generated and/or analyzed during this study are available from the corresponding author upon reasonable request.
